# Stomal variceal haemorrhage in ileal conduit diversion: a rare case report and literature review

**DOI:** 10.3389/fonc.2024.1440828

**Published:** 2024-10-24

**Authors:** Mingjie Xu, Yiwei Lin, Bohua Shen, Geming Chen

**Affiliations:** Department of Urology, The First Affiliated Hospital, Zhejiang University School of Medicine Hangzhou, Zhejiang, China

**Keywords:** bladder cancer, ileal conduit, stomal varices, haemorrhage, interventional radiological management

## Abstract

**Background:**

Radical cystectomy is typically recommended for managing bladder cancer, with ileal conduit diversion being a prevalent form of urinary diversion. Stomal variceal haemorrhage is a rare complication of ileal bladder diversion and poses diagnostic and therapeutic challenges that can escalate to life-threatening circumstances. Hepatic cirrhosis and cancer liver metastasis-induced portal hypertension are considered the main causes of stomal varices. However, the real-world expertise in the pathophysiology of, diagnostic approach to, and overall management strategy for stomal variceal haemorrhage in ileal conduit diversion is limited.

**Case presentation:**

Herein, we present a rare case of a 77-year-old man with bladder cancer who developed stomal variceal haemorrhage after undergoing radical cystectomy and ileal conduit urinary diversion. Imaging revealed that the peristomal varices communicated with the subcutaneous veins of the abdominal wall without apparent portal hypertension. Transhepatic coil embolization of bleeding stomal varices was successfully performed via a transhepatic antegrade approach. No complications or stomal variceal haemorrhage occurred during a 6-month follow-up period.

**Conclusion:**

Transhepatic coil embolization may be considered an initial therapeutic option for patients who experience stomal variceal haemorrhage. Comprehensive management of underlying liver disease and portal hypertension is needed at follow-up visits. We describe successful experience with the precise treatment of this rare and atypical disease, conduct a thorough review of the pertinent literature, and deliberate on optimized diagnostic and therapeutic procedures.

## 1 Introduction

Radical cystectomy is the standard treatment for muscular invasive bladder cancer and some high-risk non-muscular invasive bladder cancer (1). The ileal bladder is a type of standard urinary diversion for patients undergoing radical cystectomy that is relatively safe and widely applied (2, 3). Postoperative complications of ileal conduits include parastomal hernias, bowel obstruction, stomal necrosis, internal hernias, peristomal dermatitis, and anastomotic stricture (4–6). Stomal variceal haemorrhage is a rare complication of ileal conduit urinary diversion that occurs primarily in patients with portal hypertension resulting from hepatic cirrhosis or liver metastases (4, 7, 8). Bleeding of peristomal varices can sometimes lead to a life-threatening situation (8).

This article reports a case of ileal conduit diversion-associated stomal variceal haemorrhage without visible superficial peristomal varices or clinical portal hypertension. The haemorrhage was effectively controlled by transhepatic coil embolization without any potential intra- or postoperative complications. The atypical clinical symptoms of the patient, along with the rare and successful use of transhepatic coil embolization therapy in treating stomal variceal haemorrhage in ileal conduit diversion, enhance the distinctive nature of this case.

## 2 Case presentation

**Figure 1:**
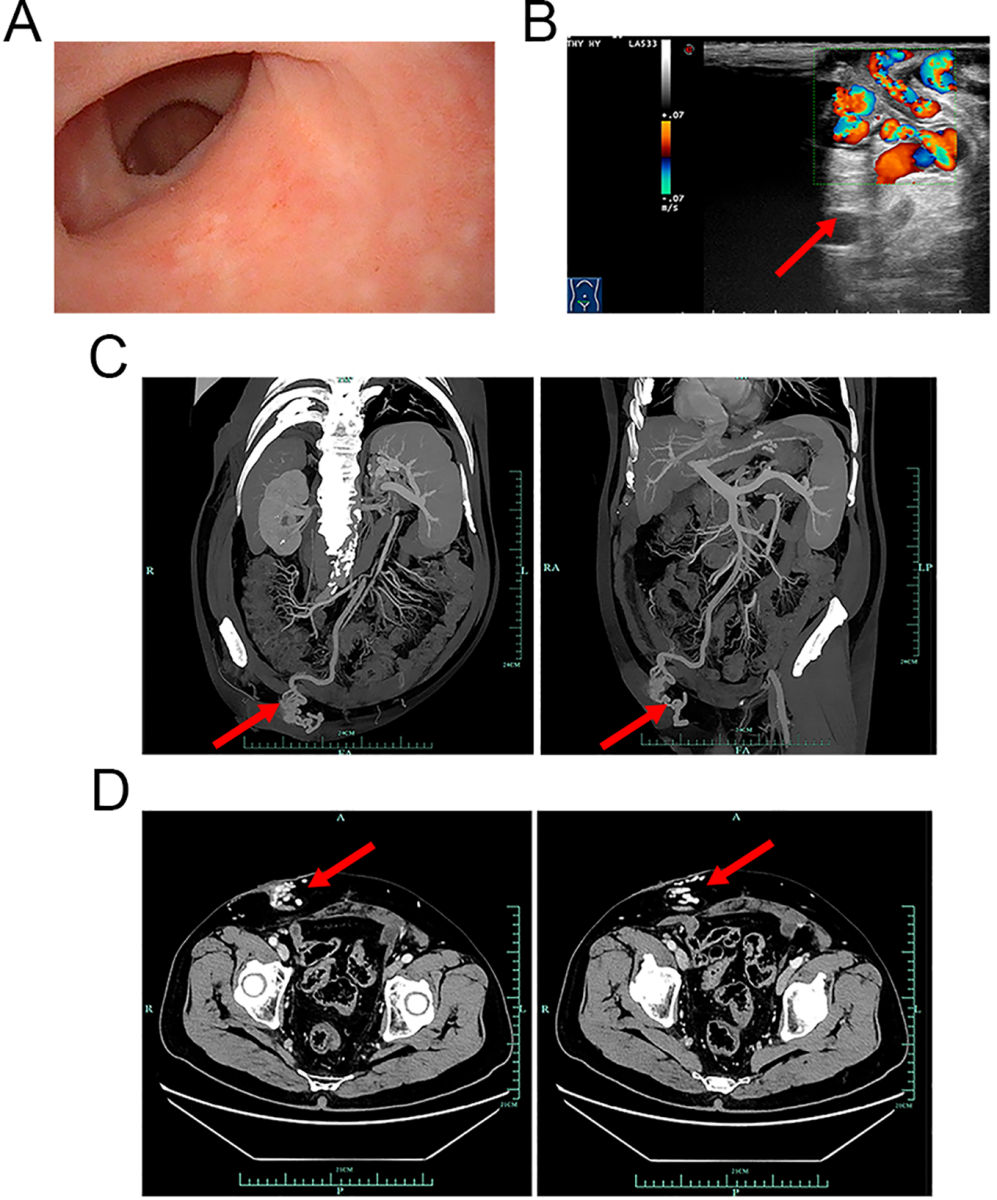
Cystoscopy, ultrasonic, and CTA examination images. **(A)** Cystoscopy revealed that the mucosa of the ileal conduit was smooth. No obvious varicose veins, bleeding points, or tumour recurrence was observed. **(B)** CFDI showing that the vessels around the vesicostomy are tortuous, extending upwards to the mesenteric vessels and downwards to the iliac veins. Local luminal blood flow signals were abundant, with a flow velocity of 20 cm/s (red arrow). **(C, D)** CTA images of the mesenteric artery showing that the varicose veins around the stoma were connected to the subcutaneous veins of the abdominal wall (red arrows). **(C)** Coronal and sagittal planes. **(D)** Cross-section. CTA, computed tomography angiography; CFDI, colour flow Doppler imaging.

The patient was a 77-year-old Asian man. In November 2023, he presented to The First Affiliated Hospital, Zhejiang University School of Medicine, with an episode of painless gross haematuria for 1 day. He had undergone postradical cystectomy with an ileal conduit because of bladder cancer in July 2008. According to the American Joint Committee on Cancer (AJCC) staging system, the disease status of bladder cancer in this patient was T_2_N_0_M_0_ or stage I at that time. Posttreatment computed tomography (CT) results were stable, with no evidence of active disease at the resection cavity. Previously, he had medically controllable high blood pressure and type II diabetes for more than 10 years, with no tobacco or alcohol addiction. He had no history of hepatitis, cirrhosis, or other liver dysfunction. The patient had no history of melena or any other gastrointestinal disorder. He denied having a genetic history or a similar history in his family. A physical examination revealed gross haematuria in the ostomy bag. There were no redness, pus, or visible superficial varices around the stoma. The abdomen and kidney areas were without percussion pain. Laboratory evaluation revealed that the viral hepatitis test was negative, and the serum α-fetoprotein level was normal. The serum electrolytes and liver function indicators were within normal limits, but the creatinine level was 143 μmol/L (normal range, 57–111 μmol/L). The complete blood count (CBC) revealed that the haematocrit level was 30.6% (normal range, 38%–50.8%), the haemoglobin level was 101 g/L (normal range, 131–172 g/L), the white blood cell (WBC) count was 4.98 × 10^9^/L (normal range, 4.0–10.0 × 10^9^/L), and the platelet count was 149 × 10^9^/L (normal range, 83–303 × 10^9^/L). The prothrombin time was 11.6 seconds (normal range, 10.0–13.5 seconds), and the partial thromboplastin time was 27.2 seconds (normal range, 23.9–33.5 seconds). A urine test was positive for red blood cells (RBCs), WBCs, and leukocyte esterase. No clear signs of neoplasm recurrence or urinary calculi were identified on the chest or abdomen CT images. Cystoscopy revealed no signs of apparent bleeding points, superficial varicose veins, or neoplasm recurrence (**Figure 1A**).

To determine the cause of the haemorrhage, additional imaging examinations were performed. Ultrasonic examination revealed indications of a fatty liver and an enlarged spleen. The portal veins were not significantly dilated; the inner diameter of the main portal vein was 11 mm, that of the right portal vein was 10.1 mm, and that of the left portal vein was 9.7 mm. The flow velocity of the portal veins was within the normal range; that is, that of the main portal vein was 30.4 cm/s, that of the right portal vein was 21.6 cm/s, and that of the left portal vein was 22.7 cm/s. Interestingly, despite the absence of visible superficial peristomal varices during physical examination and cystoscopy, ultrasonic examination revealed that vessels around the stoma were tortuous and connected to the mesenteric and iliac veins (**Figure 1B**, red arrow; the flow velocity was 20 cm/s). Computed tomography angiography (CTA) examination of the mesenteric artery confirmed that peristomal varices communicated with the subcutaneous veins of the abdominal wall (**Figures 1C, D**, red arrows). Therefore, it was concluded that the patient’s haematuria was due to a haemorrhage from ileal conduit diversion-associated stomal varices.

**Figure 2:**
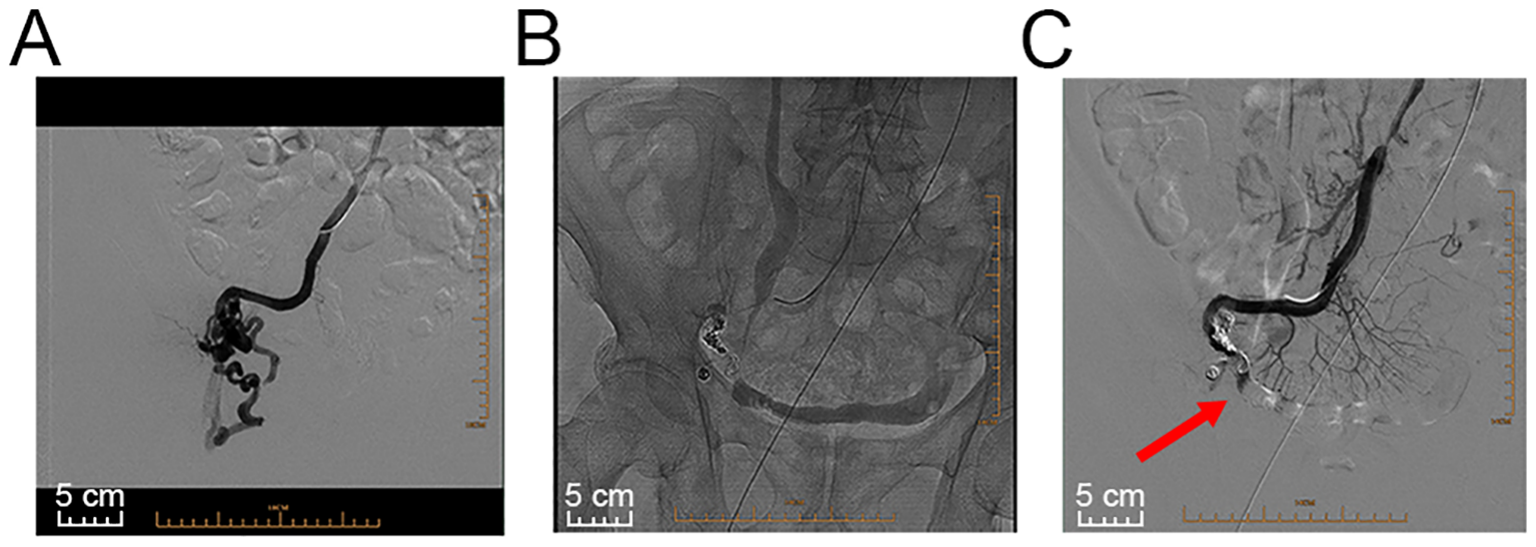
Images of interventional radiological management. **(A)** Angiography revealed stomal and pelvic varices. **(B)** A catheter was used to perform coil embolization of the main feeding ectopic varices and subsequent coil embolization of the access vein and smaller stomal varices. **(C)** Angiography confirmed complete occlusion of the target varicose veins (red arrow).

**Figure 3:**
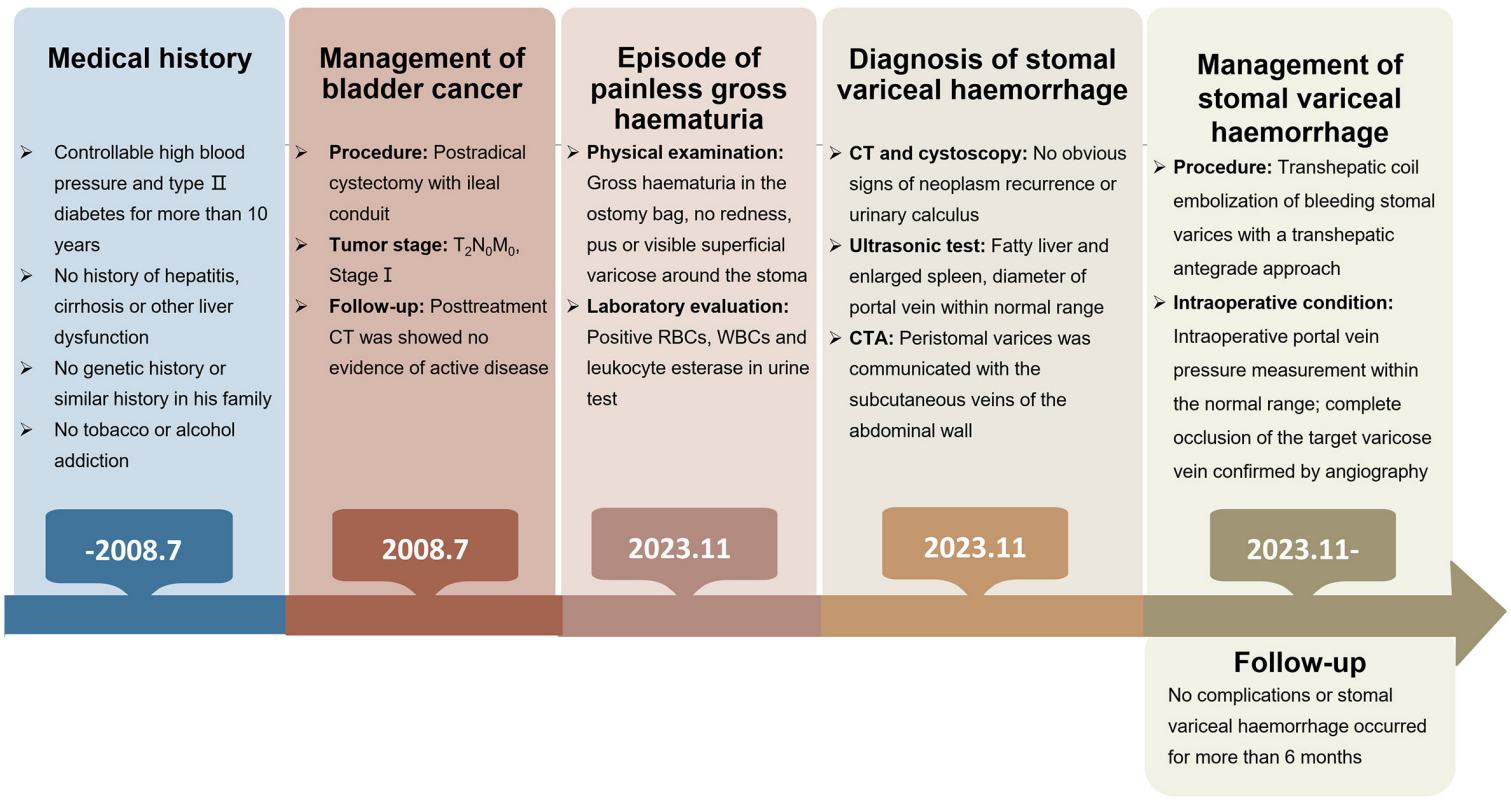
A brief description of the diagnosis and treatment of the present patient.

With the consent of the patient, interventional radiological management of local stomal varices was conducted to achieve better control of bleeding (**Figure 2**). First, ultrasound-guided percutaneous puncture of the branch of the left intrahepatic portal vein was performed, which was traversed with a catheter. Angiography revealed apparent peristomal and pelvic varices, and the main branches of the portal vein were visible (**Figure 2A**). The traversed catheter was then used to perform coil embolization of the main feeding ectopic varices (**Figure 2B**). Intraoperative portal vein pressure measurements were within the normal range. Finally, angiography confirmed complete occlusion of the target varicose veins (**Figure 2C**, red arrow). After the procedure, the haemorrhage was effectively controlled, with no potential intra- or postoperative complications for more than 6 months. The follow-up of the patient continued. **Figure 3** provides a brief description of the diagnosis and treatment of this patient.

## 3 Discussion

Ectopic varices are dilated portosystemic collateral veins at unusual sites. Ectopic varices account for 1%–5% of all variceal bleeding but are rarely reported in ileal conduit stomas (9, 10). To our knowledge, only 27 such patients, including the presented patient, have been reported in the accessible literature (7, 8, 10–25). The mean age of the 23 men and 4 women was 66.07 years. The first report of stomal variceal haemorrhage in patients with ileostomies was published in 1968 by Resnick et al. (26). Bleeding from stomal varices in an ileal conduit was first described by Foulkes and Wallace in 1975 (27). In a comprehensive analysis of 244 patients without a history of liver disease who were followed up for 9 years after the establishment of an ileal conduit, only 1.5% experienced stomal haemorrhage as a delayed complication (5). The pathogenesis of ileal conduit diversion-associated stomal varices is mainly considered to include portal hypertension, which is caused mostly by liver cirrhosis or cancer liver metastasis. Additional potential causes require further evidence-based medical verification. For instance, mesenteric abnormality is hypothesized as a potential cause in patients with unexplained stomal variceal haemorrhage (12). Stomal haemorrhage can also be the first manifestation of hepatic cirrhosis (21). These varices are formed at the mucocutaneous border of the stoma because of the connection between the high-pressure portal system and the low-pressure venous network of the abdominal wall (21). Furthermore, the blood flow in the ileum near the portal region is susceptible to the influence of portal pressure. Thus, patients with preclinical portal hypertension, despite the absence of apparent cirrhosis indications, may develop ileal stomal varices (19, 28). The interval for the development of ectopic varices and subsequent haemorrhage may be affected by the severity of the underlying liver disease and has been reported to range from 1.5 to 348 months (22). In our patient, no abnormal liver functions were detected by biochemical examination, and both the abdominal CT and ultrasonic examinations revealed no signs of cirrhosis or tumour metastases. Intraoperative measurements of portal vein pressure were within the normal range. Thus, these findings led to a negative assessment of portal hypertension. Nevertheless, the abdominal CT revealed splenomegaly, which is a symptom of preclinical portal hypertension. This symptom could be attributed to regional portal hypertension (29) or preclinical cirrhosis. Unfortunately, the patient refused further invasive examinations to identify the root cause of splenomegaly.

The diagnosis of stomal variceal haemorrhage in ileal conduit diversion may be overlooked or delayed. A history of liver cirrhosis and cancer liver metastasis and a physical examination of caput medusae should alert clinicians to the presence of parastomal varices. However, at times, peristomal varices may not be clinically evident during physical examination, similar to those in our patient. Other causes of urinary tract haemorrhage, such as genitourinary cancer and urinary obstruction/calculi, should be considered when clinical symptoms are atypical (14). In some cases, the presence of varicose veins in deep regions of the stoma can be evaluated via endoscopy. The reconstruction of CTA angiograms significantly promotes an understanding of the variceal pathways and abnormal anatomical connections (16). Mesenteric angiography during the venous phase is able to detect varicose veins (19). In situations involving compromised kidney function in which intravenous contrast is not recommended, retrograde ureteral probing with contrast agent visualization and vascular ultrasonic examination can be considered to clarify the diagnosis (14). In conclusion, a thorough diagnosis necessitates a meticulous evaluation of the mucocutaneous region surrounding the stoma and an endoscopic inspection of the ileal conduit. In our particular scenario, CTA reconstruction allowed for accurate visualization of the paths of ileal varices, facilitating the effective planning of therapeutic interventions by urologists and radiologists.

Although a variety of therapeutic options have been advocated for, there is no consensus on the optimal treatment for stomal variceal haemorrhage in ileal conduit diversion. Local treatment options, such as pressure, cautery, sclerotherapy, and suture ligation, have the potential to be effective, at a much lower cost than surgical intervention (10, 15, 19). However, these treatment options have limitations due to complications such as necrosis and perforation, as well as a high risk of massive haemorrhage after the sloughing of occluded varices (19). Interventional radiological obliteration (including coils, glues, particles, thrombin, or *N*-butyl cyanoacrylate) is another therapeutic option for ileal conduit diversion-associated stomal variceal haemorrhage (16, 30, 31). A recent review revealed that the technical success rate of embolization of parastomal and ectopic small bowel varices via a transhepatic antegrade approach was 100% (n = 17), and the early clinical success rate was 82.3%. No patient experienced complications (n = 14) (30). Nevertheless, in patients who present with evident portal hypertension and repeated bleeding despite interventional radiological obliteration or local therapies, reducing portosystemic pressure is a primary goal for treating haemorrhagic episodes. The therapeutic options vary from mucocutaneous disconnection, a transjugular intrahepatic portosystemic shunt (TIPS), to portosystemic shunting procedures (7, 30, 32). Notably, there are individuals who are not eligible candidates for TIPS or shunting procedures because they are in an end-stage condition or experience hepatic encephalopathy (13). In addition, conservative management of underlying liver disease and portal hypertension is needed. β-Blockers, somatostatin analogues, and vasopressin have been used to reduce portal hypertension (33). Our multidisciplinary team (MDT) concluded that reducing portal vein pressure via TIPS or shunting procedures was not suitable for the patient and that sclerotherapy or variceal ligation may be ineffective because of the fast flow velocity of local varicose veins (20 m/s). Thus, transhepatic coil embolization of bleeding stomal varices was successfully performed via a transhepatic antegrade approach (**Figure 2**). Notably, the patient in the present case needs long-term follow-up for better surveillance of stomal variceal haemorrhage, liver function, and portal vein pressure.

**Figure 4:**
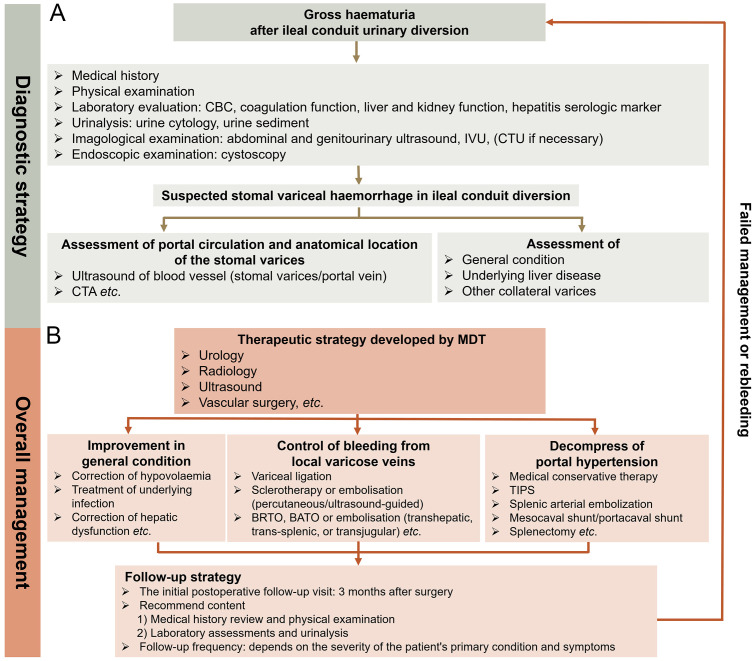
Diagnostic strategy and comprehensive management of stomal variceal haemorrhage in ileal conduit diversion. **(A)** Diagnostic strategy. Accurate diagnosis of stomal variceal haemorrhage involves ruling out other common conditions (especially genitourinary cancer and urinary obstruction/calculi) that may lead to haematuria and identifying potential portal hypertension. If ultrasound, cystoscopy, and IVU fail to pinpoint the cause of haematuria, a CTU examination is recommended. Utilizing reconstructed CTA provides precise visualization of the course of ileal varices, allowing urologists and radiologists to plan therapeutic interventions effectively. In cases indicating signs of portal hypertension, it is crucial to investigate the presence of varices in collateral venous pathways of the portal system, particularly oesophageal and gastric varices. **(B)** Comprehensive management. It is advisable for an MDT to collaborate in developing treatment strategy. The MDT should comprise experts in urology, radiology, ultrasound, and vascular surgery, with potential involvement in general surgery and hepatology as needed. Therapeutic decisions should be based on understanding the anatomical location of stomal varices, the severity of underlying liver disease, and a thorough assessment of the patient. Local therapeutic options such as cyanoacrylate injection, thrombin injection, and variceal band ligation, along with interventional radiological procedures like BRTO, BATO, and embolization (transhepatic, transjugular, and trans-splenic), are considered first-line treatments. Decompressive therapy for portal hypertension hinges on the anatomy and patency of the portal vascular circulation and severity of portal hypertension. Portosystemic shunting surgeries or splenectomy for treating stomal varicose haemorrhage are warranted only in highly selective cases where local and interventional radiological approaches have proven ineffective or are technically unfeasible. The initial postoperative follow-up visit is advised to take place 3 months after the surgery and should include a comprehensive medical history review, physical examination, laboratory assessments, and urinalysis. The frequency of subsequent follow-ups depends on the severity of the patient’s primary condition and symptoms. Patients must be informed to promptly seek medical attention if recurrent bleeding occurs. IVU, intravenous urography; CTU, computed tomography urography; CTA, computed tomography angiography; MDT, multidisciplinary team; BRTO, balloon-occluded retrograde transvenous; BATO, balloon-occluded antegrade transvenous obliteration.

The comprehensive management of rare complications of the procedures used presents a challenge because there is insufficient evidence to guide therapeutic decisions. Owing to the low occurrence and variability in variceal anatomy, large randomized controlled trials of ileal conduit diversion-associated stomal variceal haemorrhage are limited. Therefore, sharing real-world experiences through case reports is crucial to ensure that emerging techniques can be appropriately applied in the context of stomal variceal haemorrhage in ileal conduit diversion. Based on previous studies (14, 32) and the experience of our centre, herein, we discuss a diagnostic strategy and overall management for ileal conduit diversion-associated stomal variceal haemorrhage and propose an innovative clinical procedure for improving clinical outcomes (**Figure 4**). Considering its clinical efficacy and cost-effectiveness, our centre is gathering more experience with ileal conduit diversion-associated stomal variceal haemorrhage to further optimize this clinical diagnostic and therapeutic procedure.

## 4 Conclusion

Stomal variceal haemorrhage must be considered in patients with ileal conduits and repeated occurrences of haematuria, especially in those with portal hypertension. However, sometimes, the clinical symptoms of patients are atypical, which requires that practitioners have more extensive experience to make accurate diagnoses. This work describes a patient who developed ileal conduit diversion-associated stomal variceal haemorrhage without clinical portal hypertension. Transhepatic coil embolization may be considered an initial therapeutic option for patients who experience stomal variceal haemorrhage in ileal conduit diversion. After percutaneous embolization, comprehensive management of underlying liver disease and portal hypertension is needed at follow-up visits. Optimized diagnostic and therapeutic procedures are proposed to improve the clinical outcomes of stomal variceal haemorrhage in ileal conduit diversion.

## 5 Patient perspective

During the phone follow-up on May 1, 2024, the patient expressed satisfaction with the transhepatic coil embolization of the bleeding stomal varices. The procedure not only led to good efficacy with mild pain but was also cost-effective. The patient maintained good physical condition without any complaints of haemorrhage postprocedure.

## Data Availability

The original contributions presented in the study are included in the article/**Supplementary Material**. Further inquiries can be directed to the corresponding author.
