# Supplementary material for: Stomal variceal haemorrhage in ileal conduit diversion: a rare case report and literature review
Source: Front Oncol. 2024 Oct 24;14:1440828. doi: 10.3389/fonc.2024.1440828 (PMC11540816; doi:10.3389/fonc.2024.1440828)
Supplement: Supplementary file 1 [file Table1.docx]

| **Publication year** | **Gender** | **Number of cases** | | **Age^*^** | **Reference** |
| --- | --- | --- | --- | --- | --- |
| 1997 | Male | 1 | 52 | | 11 |
| 1997 | Male/Female | 13^**^ | 63.7 | | 22 |
| 2000 | Male | 2 | 68 | | 14 |
| 2005 | Male | 1 | 68 | | 12 |
| 2007 | Male | 1 | 71 | | 7 |
| 2010 | Male | 1 | 68 | | 13 |
| 2012 | Male | 1 | 63 | | 20 |
| 2013 | Female | 1 | 70 | | 15 |
| 2014 | Male | 1 | 60 | | 16 |
| 2014 | Male | 1 | 68 | | 19 |
| 2015 | Male | 1 | 72 | | 8 |
| 2016 | Male | 1 | 74 | | 10 |
| 2018 | Female | 1 | 77 | | 25 |
| 2024 | Male | 1 | 77 | | The presented patient |

**Supplementary Table**

*For more than one patient, the mean age is presented.

**Comprising 11 males and 2 females.
